# Contextualised physical metrics: The physical demands vary with phase of play during elite soccer match play

**DOI:** 10.1002/ejsc.12209

**Published:** 2024-10-27

**Authors:** Benjamin W. C. Jerome, Michael Stoeckl, Ben Mackriell, Christian W. Dawson, Daniel T. P. Fong, Jonathan P. Folland

**Affiliations:** ^1^ School of Sport Exercise and Health Sciences Loughborough University Loughborough Leicestershire UK; ^2^ Stats Perform Chicago Illinois USA; ^3^ Department of Computer Science Loughborough University Loughborough Leicestershire UK

**Keywords:** phase of play, physical demands, soccer

## Abstract

The physical demands of elite soccer match play have traditionally been measured using aggregated whole‐match metrics. However, match play is increasingly considered as distinct phases of play, although the influence of phase of play on match physical demands remains largely unknown. This study compared physical intensity, acceleration and deceleration demands, between phases of play and according to playing position. The duration of each match from a major European league (*n* = 1083) was divided into one of five reciprocal phases (for the in‐/out‐of‐possession team) using event and tracking data: build‐up/high‐block, progression/mid‐block, chance creation/low‐block, fast attack/fast defence, or attacking transition/defensive transition. Player tracking data were used to calculate physical intensity as the rate of distance covered (m⋅min^−1^) in total and within five speed categories, and the proportion of time spent accelerating and decelerating (>2 m s^−2^) during each phase of play. Rate of distance covered in total differed markedly with phase of play; fast attack 35%–53% greater, and fast defence 33%–50% greater, than other in‐/out‐of‐possession phases respectively, and these effects were amplified for the rate of distance covered at higher speeds (e.g., sprinting ≥4‐fold differences between phases). Match phase also affected the proportion of time spent accelerating and decelerating (highest for fast attack and chance creation, respectively), especially when in‐possession for forwards and when out‐of‐possession for defenders (*p* < 0.001). Phase of play had a large effect on the physical intensity of match play, particularly rates of distance covered at higher speeds, as well as the acceleration and deceleration demands, and in a position specific manner.

## Highlights

- Despite elite soccer match play being increasingly considered as distinct phases of play, the effect of phase on physical metrics is largely unknown. This analysis of 1083 matches of a major European league found rates of distance covered in total, as an index of physical intensity, differed markedly (by >50%) according to phase, with even more pronounced effects on the rates of distance covered at higher speeds (e.g., sprinting >4‐fold greater for fast attack and fast defence than some phases).
- Playing position also had a pronounced interaction with phase of play; fast attack (forwards) and fast defence (midfielders and defenders) phases involving considerably greater physical intensities. Moreover, phase of play also had a large effect on the proportion of time spent accelerating and decelerating, most notably when in‐possession for forwards and when out‐of‐possession for defenders.
- Thus, the present study is the first to comprehensively divide the entire duration of match play into distinct phases using event and tracking data, demonstrating that match physical demands during elite soccer match play are extensively effected by match phase, and in a position specific manner.

## 1 INTRODUCTION

Physical metrics during elite soccer match play have traditionally been measured over the whole match, which may give some insight into the overall physical demands (i.e., volume of physical work) but provides no indication of physical intensity or how intensity changes according to the varying context throughout a match. Specifically, match play is increasingly being broken down or contextualised into different phases of play, such as attacking, defensive and transition phases, or more specific phases (e.g., FIFA phases of play) (Bortnik et al., 2022; Bradley, 2023). However, the influence of phase of play on match physical intensity is largely unknown. A holistic examination of match play which quantifies the physical metrics, particularly intensity, during different phases of play may advance our understanding of physical metrics in elite soccer.

Soccer match play is highly dynamic and involves a series of collective movement patterns, typically referred to as phases of play (Bauer et al., 2023; Sampaio et al., 2012). However, research to date has typically reported physical demands as aggregate whole match metrics (Bradley et al., 2018) with little known about how physical metrics change during different phases of match play. It is reasonable to assume that the physical intensity of match play, assessed as the rate of distance covered, varies with the phase of play although this has not been rigorously examined. For example, phases which consist of slow and controlled possessions within a team's defensive half of the pitch (e.g., ‘build‐up’) may be expected to involve predominantly low‐to‐moderate intensity movement, whereas phases which involve a turnover in possession (‘attacking transition’/‘defensive transition’) or rapid movement up/down the pitch (‘fast attack’/‘fast defence’) may involve higher intensity. Two previous studies of a limited number of matches (*n* = 10) of a single team indicated that a range of physical metrics vary between some specific phases of play (Bortnik et al., 2022, 2023).

Furthermore, accelerations and decelerations which occur during match play involve distinct physiological and biomechanical demands (Caldbeck et al., 2022; Dalen et al., 2016; Harper et al., 2019; Varley et al., 2017). For example, acceleration involves a high rate of muscular work and metabolic stress (Hader et al., 2016), whereas deceleration involves high musculoskeletal loading, a likely elevated risk of injury (e.g., anterior cruciate ligament injuries) (Alentorn‐Geli et al., 2009; Cochrane et al., 2007; Harper et al., 2022; Johnston et al., 2018) and eccentric contractions that initiate muscle damage leading to delayed onset muscle soreness (Lieber, 2018). As such, acceleration and deceleration are important physical metrics that are frequently measured during training and match play (Akenhead et al., 2016). Phases of play, which involve short‐sharp movements and frequent changes in direction (e.g., attacking/defensive transition), likely involve more time spent accelerating and decelerating, compared with phases that typically involve slower passing/movement of the ball (e.g., build‐up/high‐block). For example, two small studies (*n* = 10 matches) have shown that the number of accelerations and decelerations per minute differed considerably according to the phase of play (Bortnik et al., 2022, 2023). Overall, a large‐scale study which compares the rates of distance covered (m·min^−1^) in total and within different speed categories, as well as the acceleration and deceleration demands, between different phases of play during a match has yet to be completed and may reveal the full extent of how physical metrics are affected by the phase of play.

Possession status has been found to effect match physical intensity, with higher rates of distance covered in total (+7.8%), and especially within the running (+31%) and high‐speed (+30%) categories, when teams are out‐of‐possession versus in‐possession (Jerome et al., 2023). Therefore, a comprehensive approach would seem to include the effects of phase of play on physical demands while considering a team's possession status. Playing position is also known to effect the physical intensity of match play (Barnes et al., 2014; Bradley et al., 2009), and therefore, it is possible that phase of play interacts with playing position to effect the physical intensity of any given player (Bortnik et al., 2023). For example, forwards and defenders may have greater physical intensity (rates of distance covered) and acceleration and deceleration demands during attacking (in‐possession) and defensive (out‐of‐possession) phases, respectively, whereas midfielders may have high physical demands in both, although this has yet to be fully explored. Collectively, such an analysis may provide unique insights into how physical demands are influenced by the match phase, allowing coaches and practitioners to develop contextualised physical preparation strategies.

The aims of this study were: firstly, to compare the physical metrics, particularly rates of distance covered as a measure of intensity, and the acceleration and deceleration demands, between five distinct reciprocal (with respect to the in‐possession/out‐of‐possession teams) phases of match play, specifically build up/high‐block, progression/mid‐block, chance creation/low‐block, fast attack/fast defence, and attacking transition/defensive transition; secondly, to examine position‐specific differences in the physical metrics, and the acceleration and deceleration demands, according to phase of play. It was hypothesized that: physical intensity would vary with the phase of play and according to playing position, with fast attack phases involving higher physical intensity than build‐up phases; the proportion of time spent accelerating and decelerating would be the greatest during attacking and defensive transition phases, which may involve short‐sharp movements and frequent changes of direction.

## 2 METHODS

### 2.1 Raw data collection

On‐ball event and player tracking data for 1,083 matches of a major European league were collected by *Stats Perform LLC* (Stats Perform, Chicago). Event data were manually coded by the data provider at a frame rate of 25 Hz providing details of the technical event (e.g., pass, shot, and free‐kick) and active player interacting with the ball, which were subsequently used to determine whether the ball was in play (or not) and which team had possession at any given instance. The inter‐operator reliability of the company's ball event data collection system has been verified (Liu et al., 2013). Individual player tracking data were collected by a real‐time optical tracking system (Stats Perform's SportVU version 2.12.0, three‐camera BASLER acA2500‐14gc, 2,560 × 1,500 pixels HD system, 16 frames/s), which provides x‐ and y‐coordinates for all players and the ball. This tracking system has been validated for tracking player movement compared to a gold‐standard system (Vicon Motion Capture; root mean square measurement error: 56 ± 16 cm) (Linke et al., 2018). Raw tracking data were processed by Stats Perform and individual player position, speed and acceleration/deceleration values were provided at a sampling frequency of 10 Hz. Ethical approval was granted by the University's ethics review subcommittee.

For the comparison of different phases of play, ball‐in‐play (effective playing) time during each match was divided into five reciprocal (with respect to the in‐possession/out‐of‐possession teams) phases of play: build‐up/high‐block, progression/mid‐block, chance creation/low‐block, fast attack/fast defence, and attacking transition/defensive transition (defined below and equating to 10 separate phases when considered on an individual team basis). The individual sequences labeled as a particular phase were aggregated to determine the total duration of each phase, during which match physical metrics were calculated.

### 2.2 Phases of play

Research on phases of play is relatively nascent and consequently there is no agreed consensus on standardised phases of play and their definitions within football match play. Therefore, investigating phases of play requires the creation of phases with specific definitions. The phases defined below and their definitions were developed following a review of previous research and practice (e.g., FIFA Phases of Play Metrics) (Bauer et al., 2023; Sarmento et al., 2018).

### 2.3 Build‐up/high‐block, progression/mid‐block, and chance creation/low‐block

These reciprocal phases were derived using the location of the ball (pitch length and width were standardised to a dimension of 105 × 68 m) and which team had possession. Ball possession within a team's own half was considered in the build‐up (in‐possession team) and high‐block (out‐of‐possession team) phases. Ball possession between the halfway line and halfway into the out‐of‐possession team's half was considered in the progression (in‐possession team) and mid‐block (out‐of‐possession team) phases. Ball possession between halfway into the out‐of‐possession teams half and their own (defensive) goal line was considered in the chance creation (in‐possession team) and low‐block (out‐of‐possession team) phases.

### 2.4 Fast attack/fast defence

Fast attack (in‐possession team) and fast defence (out‐of‐possession team) phases were defined as the in‐possession team progressing the ball substantially and rapidly up the pitch toward the opposition goal (>45 m of forward travel, that is, up the pitch, irrespective of ball lateral movement, in <8 s, that is, average ball speed >5.5 m⋅s^−1^). To exclude defensive clearances and misplaced passes, an additional criterion was that the in‐possession team had to have at least two interactions with the ball (labeled ‘pass’ or ‘control’ in the event data) within the opposition half of the pitch during this 8 s period (when >45 m of forward ball movement occurred). The start of this phase was the first instance when the ball was moving forward and subsequently went on to cover over 45 m in less than 8 s. The fast attack/fast defence phases ended a maximum 7 s after the first instance the 45 m had already been covered in <8 s (in order to capture actions resulting from the fast attack) if the in‐possession team retained possession, or earlier if there was a turnover in possession, or the ball went out of play within this further 7 s. When the criteria for fast attack/fast defence were met, this phase superseded all other phases of play.

### 2.5 Attacking transition/defensive transition

The attacking transition/defensive transition phases started when there was a change/turnover in possession during open play (irrespective of pitch location) and finished when there was either another change in possession, the ball went out of play, or the attacking transition team maintained possession for 6 s (in accordance with previously established definitions) (Bauer et al., 2023), at which point a new phase started. When the criteria for attacking transition/defensive transition were met, these phases superseded all other phases of play (except fast attack/fast defence).

### 2.6 Physical metrics

Physical data for each of the phases of play within a match were derived firstly for each of the outfield players of both teams, including substitutes, and secondly for the outfield players categorized according to playing position (player observations, defenders = 9,846; midfielders = 10,251; and forwards = 7,702), which was manually encoded by the data provider. Data were then averaged across both teams in the match and then averaged across all 1,083 matches analyzed. Matches with a player dismissal (*n* = 279) were excluded, as this has been shown to influence the physical metrics of players in teams with a reduced number of players (Carling et al., 2010). Also, given their positional specificity, goalkeepers were excluded from the analysis (Moreno‐Pérez et al., 2020).

### 2.7 Physical intensity

Physical intensity of each outfield player during each of the accumulated in‐possession and out‐of‐possession phases of play were calculated as follows: the rate (m⋅min^−1^) of total distance covered as well as within specific speed categories: rate of distance covered walking (0.16–1.97 m⋅s^−1^), jogging (1.98–3.97 m⋅s^−1^), running (3.98–5.47 m⋅s^−1^), high‐speed (5.48–7.0 m⋅s^−1^), and sprinting (>7.0 m⋅s^−1^) (Bradley et al., 2009; Gualtieri et al., 2023).

### 2.8 Acceleration and deceleration

Periods of sustained acceleration (>2 m⋅s^−2^) or deceleration (<−2 m⋅s^−2^) for a minimum effort duration of 0.7 s were classified as acceleration and deceleration (in accordance with previously published recommendations) (Varley et al., 2017). The end of an acceleration/deceleration effort was determined when the rate of acceleration/deceleration first went below/above 0 m⋅s^−2^ (Varley et al., 2012). Both the number of individual acceleration and deceleration periods (count) and the accumulated time spent accelerating and decelerating were calculated on a per player basis within each (accumulated) phase. The proportion of time spent accelerating and decelerating were calculated as the time spent performing that action divided by the time spent within each phase by that player.

### 2.9 Statistical analysis

The Shapiro–Wilk test was used to assess the normality of distribution and revealed that the majority of variables (≥85%) were normally distributed; therefore, parametric statistical tests were applied to provide a consistent approach. To compare match physical metrics between firstly in‐possession phases of play and then between out‐of‐possession phases of play, one‐way analysis of variance and subsequent Bonferroni post hoc tests (where ANOVA revealed significant main effects of phase) were used. Two‐way analysis of variance was also used to compare firstly the in‐possession phase and playing position effects and then the out‐of‐possession phase and playing position effects, in match physical metrics. All statistical analyses were conducted using IBM SPSS Statistics for Mac OS *X*, version 27.0 (IBM Corp.) and statistical significance was set at *p* < 0.05. All data are presented as mean ± standard deviation.

## 3 RESULTS

Total ball‐in‐play time was 57:18 ± 4:12 min:s (range 43:42–69:36 min:s) or 59.9 ± 4.8% of whole match time. As both teams in each match were part of the sample, the reciprocal (in‐possession/out‐of‐possession) phases had identical accumulated durations, with build‐up (in‐possession team) and high‐block (out‐of possession team) taking up the greatest (18.2 ± 2.2%) and fast attack (in‐possession team) and fast defence (out‐of‐possession team) the lowest (7.7 ± 1.9%; Table 1) proportions of ball‐in‐play time.

**TABLE 1:** Accumulated duration of each in‐possession and out‐of‐possession phase of play and the proportion of ball‐in‐play time taken up by each phase.

Phases of play	Total duration (min:s)	Proportion of ball‐in‐play time (%)
In‐possession		
Build‐up	10:28 ± 01:38	18.2 ± 2.2
Progression	06:53 ± 01:07	12.0 ± 1.5
Chance creation	04:04 ± 00:40	7.1 ± 1.2
Fast attack	02:12 ± 00:33	3.8 ± 0.9
Attacking transition	05:03 ± 00:47	8.9 ± 1.5
Out‐of‐possession		
High‐block	10:28 ± 01:38	18.2 ± 2.2
Mid‐block	06:53 ± 01:07	12.0 ± 1.5
Low‐block	04:04 ± 00:40	7.1 ± 1.2
Fast defence	02:12 ± 00:33	3.8 ± 0.9
Defensive transition	05:03 ± 00:47	8.9 ± 1.5

*Note*: when averaged across these matches teams experienced exactly half the reciprocal phase in each individual phase (e.g., build‐up and high block). Data are mean ± SD of 1083 matches.

### 3.1 Physical intensity during in‐possession phases of play

**FIGURE 1:**
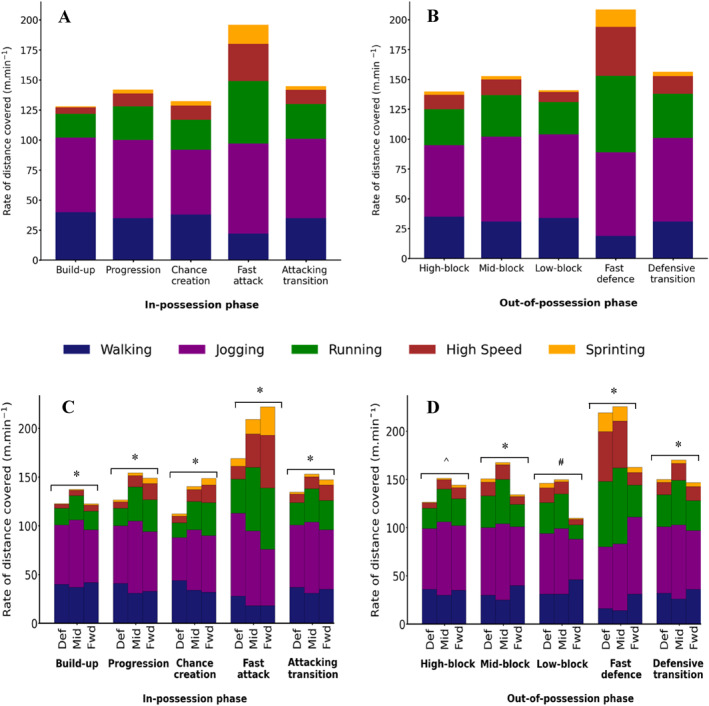
The rates of distance covered for all outfield players (A, B) and specific playing positions (C, D) during in‐possession (A, C) and out‐of‐possession (B, D) phases of play. Rates of distance covered are shown in total (whole column) and within five speed categories. Data are expressed as the average per outfield player and are the mean of 2166 team performances. Def = defenders, Mid = midfielders, and Fwd = forwards. Significant differences: * between all positions for all speed categories and in total (*p* < 0.001); ^^^ between all positions for all speed categories and in total, except jogging between defenders versus forwards (*p* < 0.001); and ^#^ between all positions for all speed categories and in total, except walking for defenders versus midfielders (*p* < 0.001).

Considering all outfield players, the rate of total distance covered differed between all the in‐possession phases (all Bonferroni *p* < 0.001; Table 2) with the lowest physical intensity during build‐up (128 ± 8 m⋅min^−1^) and with fast attack involving notably the highest intensity (>35% greater than all other phases; 196 ± 15 m⋅min^−1^; Figure 1A). Furthermore, during the fast attack phases, there were greater rates of distance covered when running (≥1.8‐fold greater), running at high‐speed (≥2.5‐fold greater), and sprinting (≥4‐fold greater) compared to all other in‐possession phases (all Bonferroni *p* < 0.001).

**TABLE 2:** Rate of distance covered during different phases of play in total and within different speed categories.

Phase of play	Rate of distance covered (m⋅min^−1^) within specific speed categories and in total					
	Walking	Jogging	Running	High‐speed	Sprinting	Total
In‐possession						
Build‐up	40 ± 3*	62 ± 6*	20 ± 4*	5.2 ± 1.5*	0.9 ± 0.5*	128 ± 8*
Progression	35 ± 3*	65 ± 6*	28 ± 5*	11 ± 3.0*	3.2 ± 1.5^†^	142 ± 10*
Chance creation	38 ± 3*	54 ± 5*	25 ± 4*	12 ± 2.7^†^	3.8 ± 1.7*	132 ± 9*
Fast attack	22 ± 4	75 ± 6*	52 ± 10*	31 ± 7.6*	16 ± 7.1*	196 ± 15*
Attacking transition	35 ± 3*	66 ± 5*	29 ± 4*	12 ± 2.7	3.2 ± 1.4	145 ± 8*
Out‐of‐possession						
High‐block	34 ± 4^	70 ± 7^#^	27 ± 6^	8.5 ± 2.6^	1.5 ± 0.7^	141 ± 10^
Mid‐block	31 ± 3^	71 ± 6^	35 ± 7^	13.0 ± 4.2^	2.8 ± 1.4^**≠**^	153 ± 12^
Low‐block	35 ± 3^	60 ± 5^	30 ± 5^	12.1 ± 3.4^	2.9 ± 1.5	139 ± 10^
Fast defence	19 ± 4^	70 ± 7^#^	64 ± 9^	41.1 ± 11.5^	14.6 ± 7.4^	208 ± 17^
Defensive transition	31 ± 3^	70 ± 5^#^	37 ± 5^	14.9 ± 3.4^	3.6 ± 1.5^	156 ± 9^

*Note*: data are mean ± SD of 2166 team performances (expressed as the average per outfield player). Significant differences: * between all other in‐possession phases (*p* < 0.001); ^†^ between all other in‐possession phases, except attacking transition (*p* < 0.001); ^ between all other out‐of‐possession phases (*p* < 0.001); ^≠^ between all other out‐of‐possession phases, except low‐block (*p* < 0.001); and ^#^ between all other out‐of‐possession phases, except fast defence and defensive transition (*p* < 0.001).

There were main effects of both playing position and phase of play, and a phase × position interaction effect, on the in‐possession rates of distance covered in total and within all the speed categories (2‐way ANOVA; all *p* < 0.001). During each in‐possession phase of play, there were also differences between all three playing positions for all physical intensity metrics (all Bonferroni *p* < 0.001; Figure 1C). Midfielders had the highest rate of total distance covered during three of the five in‐possession phases and defenders the lowest rate for all five (both *p* < 0.05). Phase had the biggest effect on the total rate of distance covered by forwards (from 123 ± 8 m⋅min^−1^ in build‐up to 222 ± 14 m⋅min^−1^ in fast attack). Furthermore, the fast attack phase was when all positions had their greatest rates of distance covered when running, running at high‐speed, and sprinting but with forwards having greater rates at higher speeds than midfielders and especially defenders (e.g., sprinting, +93% and +262%, respectively, both Bonferroni *p* < 0.001).

### 3.2 Physical intensity during out‐of‐possession phases of play

The rate of total distance covered also differed between all out‐of‐possession phases (all Bonferroni *p* < 0.001; Table 2) with the lowest physical intensity during low‐block (139 ± 10 m⋅min^−1^) and highest during fast defence (208 ± 17 m⋅min^−1^; Figure 1B). The fast defence phase had greater rates of distance covered while running (1.7–2.4‐fold higher), running at high‐speed (2.8–4.8‐fold higher), and sprinting (4.1–9.7‐fold higher) compared to all other out‐of‐possession phases (all Bonferroni *p* < 0.001).

There were main effects of both the playing position and phase, as well as a phase × position interaction effect, on the out‐of‐possession rates of distance covered in total and within all the speed categories (2‐way ANOVA; all *p* < 0.001). There were also differences between all three playing positions for most physical intensity metrics (Figure 1D). Midfielders had the highest rate of total distance covered during all five out‐of‐possession phases and forwards the lowest for four out of five phases (both *p* < 0.05). The total rate of distance covered by defenders showed the biggest effect of phase (from 130 ± 7 m⋅min^−1^ in high‐block to 220 ± 13 m⋅min^−1^ in fast defence). Consistent with the findings for all outfield players, the fast defence phase was when all positions had their greatest rates of distance covered running, running at high‐speed, and sprinting but with defenders having greater rates of distance covered at higher speeds than midfielders and especially forwards (e.g., sprinting +31% and +266%, respectively, both Bonferroni *p* < 0.001).

### 3.3 Acceleration and deceleration demands during in‐possession phases of play

There were differences between in‐possession phases for the number (count) of accelerations and decelerations, as well as the time spent accelerating and decelerating (all ANOVA *p* < 0.001; Table 3). However, even after normalization for the time spent in each phase, there were still large in‐possession phase effects for the proportion of time spent accelerating and decelerating (both *p* < 0.001). Playing position analyses focused only on the proportion of time spent accelerating and decelerating to avoid the confounding effect of phases with different accumulated durations.

**TABLE 3:** The total number (count) of accelerations and decelerations, and the total time, and proportion of time, spent rapidly accelerating and decelerating, within each phase of play.

Phase of play	Acceleration (>2 m⋅s^−2^)			Deceleration (<−2 m⋅s^−2^)		
	Count	Time (s)	Proportion of time (%)	Count	Time (s)	Proportion of time (%)
In‐possession						
Build‐up	9.2 ± 2.0*	7.4 ± 2.1*	1.7 ± 0.4*	9.7 ± 1.8*	6.1 ± 2.0*	1.3 ± 0.3*
Progression	7.9 ± 1.5*	6.2 ± 2.1*	2.2 ± 0.4*	8.8 ± 1.5*	6.7 ± 2.0*	2.3 ± 0.4*
Chance creation	6.5 ± 1.4*	4.7 ± 1.7*	2.6 ± 0.5^≠^	9.3 ± 1.5*	7.7 ± 2.8*	4.5 ± 0.7*
Fast attack	2.8 ± 0.6*	2.8 ± 1.4*	3.0 ± 0.6*	3.7 ± 0.8*	3.9 ± 1.8*	4.0 ± 0.7*
Attacking transition	6.0 ± 1.3*	5.7 ± 2.1*	2.6 ± 0.4	8.6 ± 1.6*	7.1 ± 2.7*	3.2 ± 0.4*
Out‐of‐possession						
High‐block	10.9 ± 2.6^^^	8.9 ± 2.8^^^	1.9 ± 0.5^^^	12.0 ± 2.3^^^	8.7 ± 3.2^^^	1.9 ± 0.4^^^
Mid‐block	9.4 ± 1.9^^^	6.9 ± 2.6^^^	2.3 ± 0. 5^^^	11.1 ± 1.9 ^Δ^	8.0 ± 2.6 ^Δ^	2.7 ± 0.4^^^
Low‐block	7.1 ± 1.5	5.1 ± 2.0^^^	2.8 ± 0.5	10.9 ± 1.7	8.2 ± 4.2	4.7 ± 0.7^^^
Fast defence	3.1 ± 0.7^^^	2.7 ± 1.4^^^	2.9 ± 0.6 ^Δ^	4.4 ± 1.0^^^	4.0 ± 2.1^^^	4.1 ± 0.7^^^
Defensive transition	7.2 ± 1.6 ^Δ^	6.5 ± 2.3^^^	3.0 ± 0.5^#^	8.9 ± 1.8^^^	7.9 ± 2.5 ^Δ^	3.6 ± 0.5^^^

*Note*: data are expressed per outfield player based on the average of all the outfield players of each match and mean ± SD of 1083 matches.Significant differences: * between all other in‐possession phases (*p* < 0.001); ^≠^ between all other in‐possession phases, except attacking transition (*p* < 0.001); ^ between all other out‐of‐possession phases (*p* < 0.001); ^Δ^ between all other out‐of‐possession phases, except low‐block (*p* < 0.001); and ^#^ between all other out‐of‐possession phases, except fast defence (*p* < 0.001).

**FIGURE 2:**
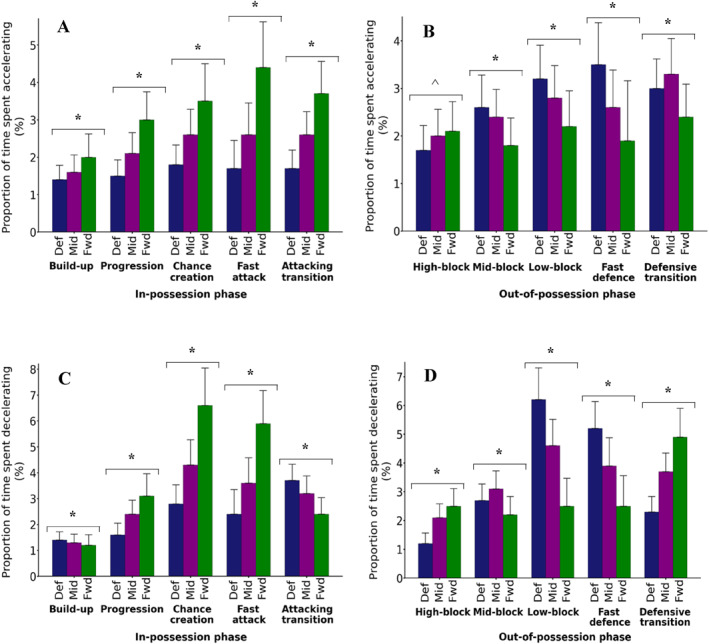
The effect of phase of play and playing position on the proportion of time spent accelerating (A, B) and decelerating (C, D) during in‐possession (A, C) and out‐of‐possession (C, D) phases of play. Data are expressed as the average per outfield player based on the average of the players in each position (defenders, Def; midfielders, Mid; and forwards, Fwd) within each match and mean ± standard deviation of 1083 matches. Significant differences: * between all playing positions (*p* < 0.001); and ^ between all playing positions, except midfielders and forwards (*p* < 0.001).

There were main effects of both phase and playing position, as well as a phase × position interaction effect, for both in‐possession acceleration and deceleration demands (2‐way ANOVA; all *p* < 0.001; Figures 2A and 2C). There were also differences in the proportion of time spent accelerating and decelerating between all three positions (all Bonferroni *p* < 0.001), with forwards having the highest proportion of time spent accelerating (5/5) and decelerating (3/5) in the majority of in‐possession phases. The effect of phase on the proportion of time spent accelerating was consistently larger in the order forwards > midfielders > defenders: defenders (from 1.4% in build‐up to 1.8% in chance creation); midfielders (from 1.6% in build‐up to 2.6% in chance creation/fast attack/attacking transition); and forwards (from 2.0% in build‐up to 4.4% in fast attack). Similarly, for the proportion of time spent decelerating, the smallest effect of phase was also for defenders (from 1.4% in build‐up to 3.7% in attacking transition), with a moderate effect in midfielders (from 1.3% in build‐up to 4.3% in chance creation), and a large effect for forwards (from 1.2% in build‐up to 6.6% in chance creation).

### 3.4 Acceleration and deceleration demands during out‐of‐possession phases of play

There were also differences between out‐of‐possession phases for the number (count) of accelerations and decelerations, as well as the time spent accelerating and decelerating (all ANOVA *p* < 0.001; Table 3). However, there were also large out‐of‐possession phase effects for the proportion of time accelerating and decelerating (both ANOVA *p* < 0.001).

There were main effects of both phase and playing position (defenders > midfielders > forwards), as well as a phase × position interaction effect, on both the out‐of‐possession acceleration and deceleration demands (2‐way ANOVA; all *p* < 0.001; and Figures 2B and 2D). During each out‐of‐possession phase there were also differences in the proportion of time spent accelerating and decelerating between all three playing positions (all Bonferroni *p* < 0.001), with defenders highest for three of the five (accelerating) and two of the five (decelerating) phases. In contrast to the findings in‐possession, when out‐of‐possession, the effect of phase on the proportion of time spent accelerating and decelerating was consistently larger in the order defenders > midfielders > forwards: forwards (from 1.8% in mid‐block to 2.4% in defensive transition); midfielders (from 2.0% in high‐block to 3.3% in defensive transition); and defenders (from 1.7% in high‐block to 3.5% in fast defence). For the proportion of time spent decelerating, there were moderate differences between phases for forwards (from 2.2% in mid‐block to 4.9% in defensive transition) and midfielders (from 2.1% in high‐block to 4.6% in low‐block) but much larger differences between the phases for defenders (from 1.2% in high‐block to 6.2% in low‐block).

## 4 DISCUSSION

The aim of this study was to provide the first comprehensive examination of the influence of phase of play on match physical metrics, particularly rates of distance covered, an index of intensity, as well as acceleration and deceleration demands, and document any position‐specific phase of play effects in a large dataset (*n* = 1,083) of elite matches. As hypothesized, physical intensity was substantially effected by phase of play, with the rate of total distance covered differing between all in‐possession and out‐of‐possession phases; lowest during build‐up and highest during fast attack when in‐possession (128 vs. 196 m⋅min^−1^) and lowest during low‐block and highest during fast defence when out‐of‐possession (139 vs. 208 m⋅min^−1^). Moreover, the effects of phase of play were increasingly pronounced for the rates of distance covered running (>1.7‐fold differences), running at high‐speed (>2.5‐fold differences), and sprinting (>4‐fold differences) being greatest during the fast attack and fast defence phases. Phase of play also had a large effect on the physical intensity of different playing positions but most notably for forwards when in‐possession (fast attack +80% vs. build‐up) and defenders when out‐of‐possession (fast defence +69% vs. high‐block). Furthermore, the proportion of time spent accelerating and decelerating was also markedly affected by both phase of play, position, and the interaction of these factors. Contrary to our second hypothesis, the highest proportion of time spent accelerating and decelerating was not typically during transition phases. The effect of in‐possession phase on the proportion of time spent accelerating and decelerating was most pronounced for forwards > midfielders > defenders, whereas the effect of out‐possession phase was reversed and tended to be larger for defenders > midfielders > forwards. The present study is the first to comprehensively divide the entire duration of match play into distinct phases using event and tracking data, demonstrating that match physical demands of elite soccer are extensively effected by the match phase and in a position specific manner.

### 4.1 Influence of phase of play on the physical intensity of match play

The present study found for the first time that the rate of total distance covered, as an index of physical intensity, differed between all in‐possession and out‐of‐possession phases, with build‐up and low‐block phases having the lowest physical intensity and fast attack and fast defence phases having the highest physical intensity (∼+50% vs. build‐up/low‐block). These differences were even more pronounced when considering the different speed categories, with the rates of distance covered running (>1.7‐fold higher), running at high‐speed (>2.6‐fold higher), and sprinting (>4.1‐fold higher) being substantially higher during fast attack (in‐possession) and fast defence (out‐of‐possession). These findings seem a logical consequence of high tempo progressive forward movement of the ball toward the defensive team's goal, and thus substantial movement of both teams up/down the pitch together, which has been previously observed (Ju et al., 2022), leading to high physical intensity during fast attack and defence phases. In contrast, the differences in physical intensity between build‐up/progression/chance creation and high‐block/mid‐block/low‐block were relatively modest (differences ≤12.5%) with predominantly low‐to‐moderate intensity movement likely due to both teams being established in their respective attacking and defensive formations (Memmert et al., 2017; Rein et al., 2017). Furthermore, the greater intensity of fast attack phase was most pronounced for forwards, whereas the greater physical intensity during fast defence was apparent for defenders, presumably due to specific positional/tactical roles (Barnes et al., 2014) and players' contesting possession in relatively large spaces. However, it is worth noting that all three positions generally had their greatest rates of distance covered at higher speeds during fast attack (in possession) and fast defence (out‐of‐possession) phases.

Interestingly, the effect of phase on physical metrics was similar for the reciprocal in‐possession and out‐of‐possession phases (i.e., greatest for fast attack/fast defence and lowest for chance creation/high block), suggesting a symbiotic relationship for the physical intensity of the two competing teams. This symbiosis was also evident when considering playing position during fast attack (highest for attacking positions) and fast defence (highest for defensive positions). This evidence supports the concept of inter‐team synchronization, whereby the behavior of the two teams is highly codependent (i.e., interrelated opposing movement patterns) (Frencken et al., 2012; Ju et al., 2022; Low et al., 2020; Sampaio et al., 2012). Thus, an original finding of this study was that match physical demands demonstrated high inter‐team synchronization during different phases of play and according to playing position.

Midfielders had the greatest physical intensity during three in‐possession and all five out‐of‐possession phases, which supports previous research (Bradley et al., 2013; Martín‐García et al., 2018; Rampinini et al., 2007) emphasizing the importance of midfielders possessing well‐developed fitness capabilities, in order to cope with the physical demands of this position. Midfielders have been shown to have greater aerobic fitness than defenders and forwards as indicated by various physiological measures such as VO_2_max (∼+12% and +4%) (Reilly et al., 2000) and running speed at anaerobic threshold (∼+7% and +17%) (Modric et al., 2020).

### 4.2 Acceleration and deceleration demands during different phases of play

The present study found that on average, each player performed ∼70 accelerations and ∼87 decelerations per match, which is similar to a previous study (Pons et al., 2021). As expected, the accumulated time spent accelerating and decelerating was higher in the phases with the longest accumulated duration during match play (Table 1). Therefore, in order to normalize for the differences in the time spent in each phase, the analysis focused on the proportion of each phase spent accelerating and decelerating. Overall, the proportion of time spent decelerating was greater than accelerating during eight out of ten in‐/out‐of‐possession phases, which highlights the prevalence of deceleration during match play (Harper et al., 2019).

Considering all outfield players, a novel finding of the current study was that the proportion of time spent both accelerating and decelerating differed between all the in‐possession and out‐of‐possession phases; being highest during fast attack (accelerating) and chance creation (decelerating) when in‐possession, and defensive transition (accelerating) and low‐block (decelerating) when out‐of‐possession. These findings were contrary to our second hypothesis, as generally the acceleration and deceleration demands were not highest during transition phases. There was also a main effect of position, such that the proportion of time spent accelerating and decelerating had opposite patterns for the in‐possession (forwards > midfielders > defenders) and out‐of‐possession phases (defenders > midfielders > forwards). Furthermore, there were marked playing position by phase of play interactions; such that the proportion of time spent accelerating and decelerating was affected by in‐possession phase of play to a variable extent, in the order forwards > midfielders > defenders; whereas, the effect of out‐of‐possession phase on the proportion of time spent accelerating and decelerating was greatest for defenders. Thus, forwards and defenders had a high proportion of time spent accelerating during fast attack and fast defence phases, respectively, which is consistent with their high rates of distance covered at higher speeds (discussed above) during these relatively brief phases (∼4% of ball‐in‐play time). These findings may be particularly important for teams that are more reliant on (fast attack) or susceptible to (fast defence) these phases and it would be advantageous for the players of these teams to possess a high capacity to accelerate and decelerate (Harper et al., 2019, 2022; Varley et al., 2012). This study also uniquely found that forwards and defenders spent a high proportion of time decelerating during chance creation (6.6%) and low‐block (6.2%), respectively, which may be indicative of frequent and sudden directional changes (involving a deceleration) as players' maneuver to find space in congested areas of the pitch (e.g., penalty box). Consequently, during these phases, these positions may be exposed to higher muscle and joint loading, as well as eccentric contractions that tend to initiate muscle damage and elevate injury risk (Dalen et al., 2016; Hader et al., 2016; Harper et al., 2019, 2022; Lieber, 2018; Varley et al., 2017). Future work examining the physical intensity associated with specific technical‐tactical actions performed during different match phases would be pertinent (Ju et al., 2023).

### 4.3 Implications for physical training and preparation strategies

The unique findings of this study can be used to inform the design of contextually specific drills, which replicate, or mimic, the physical intensities associated with different phases of play during a match. Sessions with a focus on high‐intensity aerobic and speed endurance training might incorporate physical (e.g., repeated sprints over 45 m) (Bishop et al., 2011) and/or soccer (e.g., small, medium, and large sided games) (Gaudino et al., 2014) conditioning drills that provide sufficient space/duration of physical effort for players to achieve the greater rates of distance covered during fast break/fast defence phases. Such drills may ensure optimal physical preparation (e.g., ability to perform during repeated fast attack/fast defence phases) and enable players to perform their specific tactical role/responsibilities when the attacking threat may be highest, especially toward the later stages of match play (i.e., performing under fatigue).

Furthermore, careful monitoring of acceleration/deceleration actions during training and match play has been suggested to be essential for effective player load management (Akenhead et al., 2016; Harper et al., 2019). Physical preparation strategies which condition players for position‐specific acceleration and deceleration demands during different phases may involve sided games, which elicit frequent acceleration and deceleration efforts (Hodgson et al., 2014); attacking versus defensive player drills, which replicate the movement patterns and actions of chance creation and low‐block phases; strength and conditioning for deceleration and eccentric strength, in order to habituate players to the high forces associated with sudden braking and changes of direction, and thus mitigate musculoskeletal strain and injury risk.

There are some limitations of this study. First, the accuracy of optical tracking systems to capture player displacement, and especially acceleration/deceleration (Linke et al., 2018), can vary depending on the number of cameras used, their positions, and camera specifications/settings. However, player acceleration and deceleration were determined with a minimum effort duration of 0.7 s (as recommended) (Varley et al., 2017) to avoid the error inherent within a small number of data samples, before then accumulating within each match phase, averaging across all (or position specific) outfield players within a match, and then across all matches, which may have helped to minimize random errors. Furthermore, in this study, the minimum effort duration used was based on recommendations derived using global positioning system data, although to our knowledge no equivalent for optical tracking systems has been proposed. Second, only general positional data were available and future work should consider more specific playing positions (e.g., fullback) or specialized player roles (e.g., attacking vs. defensive fullback) (Ju et al., 2023). Last, the conceptual approach and criteria used to divide match play into different phases were specific to the present study and may have influenced the derived physical metrics. Moreover, the phase definitions only included information pertaining to the ball location, although teams may be in deeper, or more advanced positions, than indicated by the location of the ball. Therefore, future studies should consider more subtle categorization of phases of play, perhaps including the collective position of the team (e.g., team centroid) and/or individual players, to more accurately capture the tactical/strategic approach of each team. However, no study to date has used both event and tracking data to comprehensively divide match play into different phases and calculated the associated physical metrics. Therefore, the present study established original criteria for defining phases of play. While these criteria could have had a minor effect, it seems unlikely that these choices had a major influence on the key findings given the pronounced magnitude of the effects identified.

## 5 CONCLUSION

This comprehensive analysis of phase of play found for the first time that match physical intensity varied markedly according to phase of play. Considering all outfield players, the rate of distance covered in total varied by >50% according to phase, with even larger effects on the rate of distance covered at higher speeds (e.g., sprinting >4‐fold differences), being greatest for fast attack and fast defence phases. Playing position also had pronounced interactions with phase of play; fast attack (for forwards) and fast defence (for defenders) phases involved considerably greater physical intensities than other phases. A further original finding was that phase of play had a large effect on acceleration and deceleration demands, most notably when in‐possession for forwards and when out‐of‐possession for defenders. The unique findings of this study can be used by coaches and practitioners to inform physical preparation strategies, such as the development of more contextually specific training drills which mimic the physical intensities, and acceleration and deceleration demands during different match phases, in order to enhance the specificity of applied practice in elite soccer.

## CONFLICT OF INTEREST STATEMENT

The authors declare that there are no conflicts of interest, that no companies or manufacturers will benefit from the results of the study, and that the results of the study are presented clearly, honestly, and without fabrication, falsification, or inappropriate data manipulation.

## Data Availability

The used data is property of Stats Perform and is not publicly available. The authors do not have permission to share the data publicly. This work can be reproduced using similar data from professional soccer (e.g., event and tracking data).
